# Census of Twitter users: Scraping and describing the national network of South Korea

**DOI:** 10.1371/journal.pone.0277549

**Published:** 2022-11-17

**Authors:** Lu Guan, Xiao Fan Liu, Wujiu Sun, Hai Liang, Jonathan J. H. Zhu

**Affiliations:** 1 School of Journalism, Fudan University, Shanghai, China; 2 Research Group of Computational and AI Communication at Institute for Global Communications and Integrated Media, Fudan University, Shanghai, China; 3 Web Mining Laboratory, Department of Media and Communication, City University of Hong Kong, Kowloon, Hong Kong, China; 4 School of Computer Science and Engineering, Southeast University, Nanjing, China; 5 School of Journalism and Communication, The Chinese University of Hong Kong, Shatin, Hong Kong, China; 6 School of Data Science, City University of Hong Kong, Hong Kong, China; King Abdulaziz University, SAUDI ARABIA

## Abstract

Population-level national networks on social media are precious and essential for network science and behavioural science. This study collected a population-level Twitter network, based on both language and geolocation tags. We proposed a set of validation approaches to evaluate the validity of our datasets. Finally, we re-examined classical network and communication propositions (e.g., 80/20 rule, six degrees of separation) on the national network. Our dataset and strategy would flourish the data collection pool of population-level social networks and further develop the research of network analysis in digital media environment.

## Introduction

Social network analysis (SNA) has been fuelling social sciences for decades. Through the use of graph theory and large network analytical techniques, the research foci of SNA differ from traditional social science regimes, shifting from social actors (e.g., individuals and organizations) to their social relationships and surrounding environments [1].

Although SNA has been employed in social media studies to examine online network patterns in the past decade, national-level population-based online social networks are still rare to find but of great importance to network studies. First, current online population networks on social media are often found to be either arbitrarily or narrowly defined, e.g., users discussing the same topics, followers of a social media account, and social media accounts of the members of an organization [2–4], whereas population-level social networks at more stable and general levels are rare to find. The causal relations and structural patterns of specific topic or event networks could hardly extend to the situations of other topics, events, or organizations. The findings relating to different topics or events are often found to result in different and even contradictory conclusions. Thus, current SNA studies require large-scale social networks at more stable community levels (e.g., nation, ethnic groups) to observe structural patterns of higher generalizability.

Second, large-scale population-based networks require precise validation methods to guarantee the completeness of datasets. Data collection of population-based networks needs to cover every actor and all their relationships in the entire population. It requires a clear boundary of the population belongingness [5]. When adopted in offline studies, the boundary is often defined according to geolocation or social identity attributes, such as students of a university or residents of a village [6]. When adopted in online studies, this network boundary becomes rather difficult due to the lack of a sampling frame for the population structure of social media users [7]. Thus, validation solutions still need to sought to ensure the data completeness.

Third, a batch of network structure propositions and social theories remain to be examined and uncovered in the national-level population social networks. Social science scholars used to rely on the national census datasets conducted by the government to capture an accurate and comprehensive overview of people’s offline lifestyle [8]. However, when depicting the online lifestyle, sampled data often become the only choice, due to the lack of sampling frames for the online population structure and the technical constrictions on data collection and storage [9]. As a result, a great number of network propositions and social science theories have few chances to be examined and summarized at the national-level social networks.

The novelty and originality of the present study lies in that, in order to enrich the national online network pools, we collected a full national social media user network, South Korea, on Twitter and proposed two validation methods to help evaluate the validity of our data collection strategy. Furthermore, to fill the research gap that online social theories were mostly examined on sampled data rather than full population network datasets, we re-examined classical network and communication propositions (e.g., 80/20 rule, six degrees of separation, etc.) on the national social network. The present study could provide practical implications by serving as a reference to help validate existing network sampling methods. Besides, the study could also bring theoretical insights by extending the classical theories contexts to online social environment.

## Related work

Previous literature on national-level Twitter accounts mainly follows two streams of data collection strategy: location-based and language-based. Bruns, Moon, Münch, & Sadkowsky scrapped the first national Twitter network in 2017 [10]. Benefiting from the comparative distinctiveness of Australian timezones and user profile geolocation tags, they successfully depicted the general network patterns of Australian “Twittersphere”. However, later, Twitter conducted a series of data protection changes to their developer APIs. Those properties, including geotags, user time zone, and the interface language used on Twitter, are all inaccessible by now [11].

The later national Twitter networks mainly utilized the language-based data collection strategy. Bruns & Enli collected the entire national Twittersphere of Norway based on the Norwegian language of user profile [12]. Bruns and others scraped the German Twitter network dataset TrISMA [13] and later the dataset served as a ground truth for the so-called rank degree sampling method [11]. Recently, Munch and Rossi compared two national Twitter networks, Italian and German, based on the language detection of user profile [14]. Our data collection mainly followed the language-based approach. Meanwhile, we also examined the geolocation information that user self-reported in their profiles. Besides, our work proposed new validations methods that could largely help validate the results.

## Materials and methods

### Ethics statements

The study was approved by the Human Ethical Review in College of Liberal Arts and Social Sciences, City University of Hong Kong. All the data collected in this study are open for the public. Data was obtained from Twitter’s REST API. Developer account was granted by Twitter before data collection started, which allows the access to the data. Identifier data fields were replaced by unidentifiable pseudo code after all data are collected upon the end of the project.

### Data collection

This current study evidenced a data collection strategy to scrape a national social media network on Twitter. The reasons we choose Twitter as the example platform are threefold. Firstly, Twitter could serve as a typical social media example, as it combines the fundamental functions of social media: both social networking services and information sharing application [15–17]. Users can both make friends with other users by following their accounts and send out their own tweets. Besides, Twitter is one of the most popular social media around the world, with 330 million monthly active users and 500 million tweets sent out per day [18]. Finally, Twitter provides advanced APIs to help researchers obtain the network data.

The reasons why we select the South Korea as the case of the nation are based on two aspects of considerations: the uniqueness of its official language and the population size of the country. Firstly, South Korea is the only country on Twitter that utilizes the Korean language as the official language. The uniqueness of its language could help us to identify the nationality of the users. Secondly, the population of South Korea was estimated at 51 million, ranking the 28^th^ in the world [19]. Compared with other countries that fit the language requirement (e.g., Japan), the population size of South Korea is more feasible for the data collection procedure.

We used Twitter’s REST APIs to collect the profiles, following relationships, and timelines of the Twitter users. The data collection of the socio-centric network requires a clear boundary to help identify the nodes within the network. In this study, we utilized the language and geolocation information that users filled in their twitter profiles to help identify the nationals of South Korean. Specifically, if the user utilized Korean as his/her language on Twitter or had filled his/her geolocation tag as South Korea or any city in South Korea, s/he will be labeled as a South Korean Twitter user. Besides, we also categorized the users according to their activeness on Twitter. Users who had updated their last posts in 2019 will be identified as active Twitter users, whereas inactive users will be excluded from our sample.

**Fig 1:**
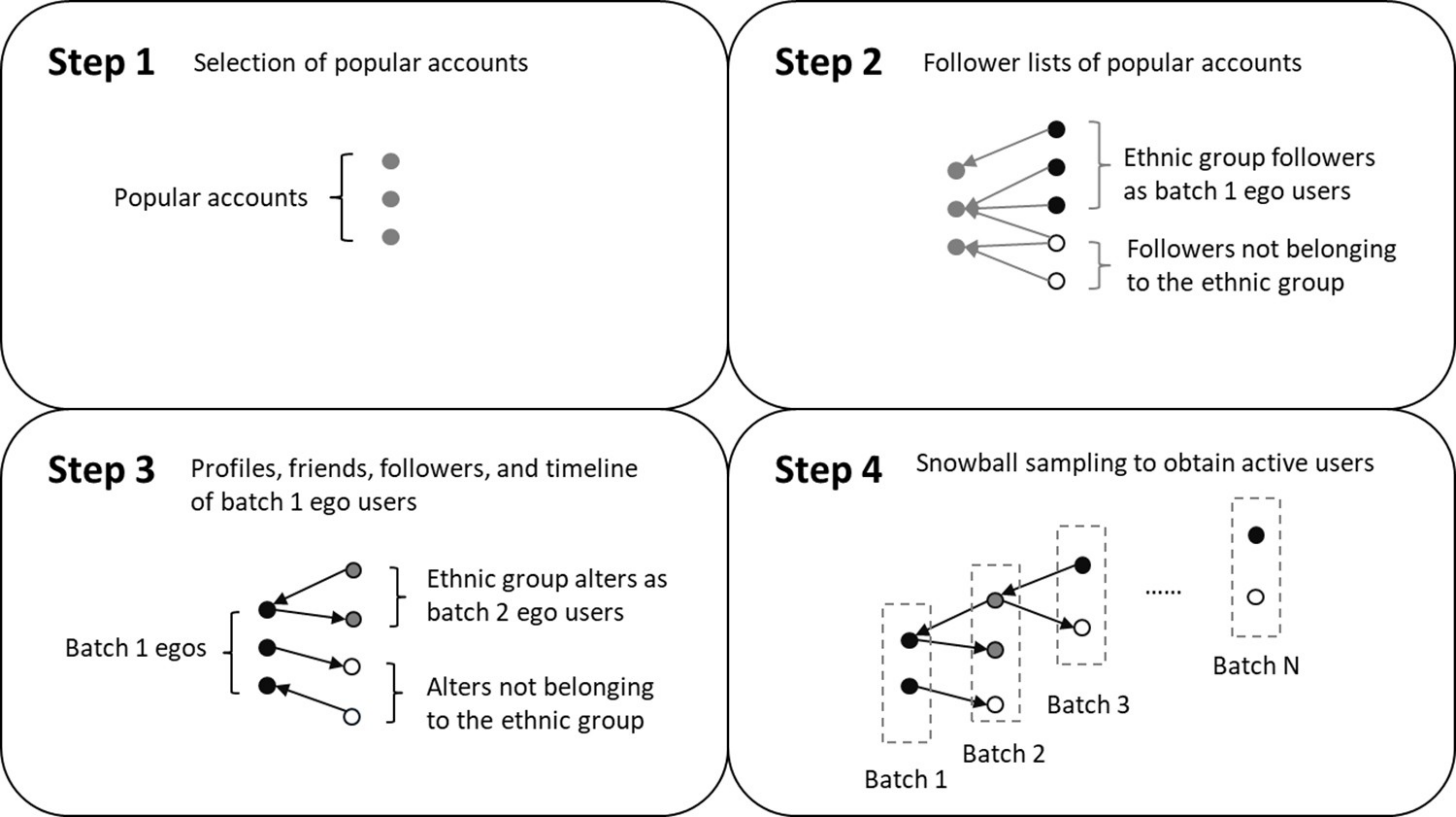
An illustration of the data collection procedure.

We utilized the snowball sampling method to collect the population of active South Korean Twitter users. Fig 1 illustrates the specific data collection procedures. In the first step, a list of the most popular Twitter accounts in South Korea was obtained from the Socialbakers’ Twitter statistics [20]. From this popularity list, 28 popular accounts in the categories of brand, society, and sports were selected as the seeds of the snowball sampling. Then, in the second step, the profiles of these popular accounts’ followers were collected through Twitter APIs. If the follower was identified as an active South Korean user in 2019, s/he will be kept as ego users in the first batch.

In the third step, we obtained the first batch ego user’s following relationship lists and tweets/ retweets in user timelines. If the alters (followers/ followees) of first batch ego users were identified as active South Korean users, they were kept and labeled as second batch ego users. By continuously repeating step 2 and step 3, we obtained profile, following relationship, and timeline data of active South Korean users until batch N.

Table 1 shows the numbers of new Twitter users, new South Korean users, and new active South Korean users scraped for each batch. After conducting six batches of snowball scraping, the increase rate of new South Korean users has declined to 1.26%. Up until the sixth batch, we obtained 127 million Twitter users’ profiles, with 18.1 million of them identified as South Korean users and 2.59 million of them as active South Korean users in 2019.

**Table 1:** Number of new users, New South Korean users, and new active South Korean users for each batch.

	New users for each batch	New South Korean users	New active South Korean users	Percentage of new South Korean active users in cumulative South Korean users
**First batch**	6,051,475	4,079,938	288,001	100.00%
**Second batch**	31,726,425	7,854,091	756,758	65.81%
**Third batch**	49,530,665	4,809,431	658,281	28.72%
**Fourth batch**	35,505,474	555,683	299,742	3.21%
**Fifth batch**	2,640,107	580,015	355,837	3.24%
**Sixth batch**	1,604,379	227,663	76,141	1.26%
**Seventh batch**	162,389	9,064	4,943	0.05%
**Eighth batch**	117,606	39,148	30,802	0.22%
**Nine batch**	133,587	28,344	12,038	0.16%
**Ten batch**	71,291	33,808	26,755	0.19%
**Eleven batch**	84,237	15,375	6,852	0.08%
**Twelve batch**	9,101	1,289	785	0.01%
**Thirteen batch**	4,165	1,887	1,536	0.01%
**Fourteen batch**	97,872	50,723	34,772	0.28%
**Fifteen batch**	60,963	10,250	3,076	0.06%
**Sixteen batch**	16,194	5,962	4,561	0.03%
**Seventeen batch**	14,379	4,428	1,632	0.02%
**Eighteen batch**	9,904	5,921	4,850	0.03%
**Nineteen batch**	6,616	2,522	1,324	0.01%
**Twenty batch**	9,151	5,543	4,503	0.03%
**Twenty-one batch**	8,103	2,372	1,354	0.01%
**Twenty-two batch**	10,873	6,057	4,808	0.03%
**Twenty-three batch**	6,907	3,245	1,938	0.02%
**Twenty-four batch**	12,126	8,523	6,064	0.05%
**Twenty-five batch**	17,779	2,971	1,604	0.02%
**Twenty-six batch**	9,254	4,946	3,945	0.03%
**Total**	127,921,022	18,349,199	2,592,902	

**Fig 2:**
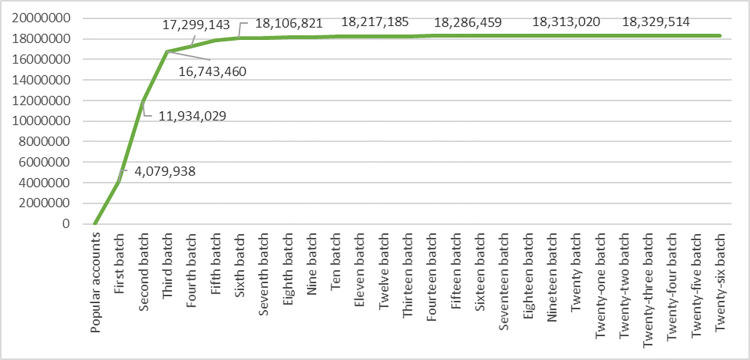
The accumulated number of active South Korean users after each batch.

To discuss the threshold for suspending the snowball scraping, we further conducted another 20 batches after the sixth batch. Fig 2 shows the accumulated number of South Korean Twitter users after each batch of snowball sampling scraping. After the third batch, the increasing trend gradually slowed down, and then the number began presenting a stable trend after the fifth batch. The increase rates after the sixth batch are all less than one percent, which is small enough to suspend the snowball scraping procedure.

### Data validation

We proposed two approaches to help evaluate the validity of our dataset. The first one is to roughly compare with public statistics of Twitter usage in South Korean. Considering the population of South Korea was estimated at 51.2 million people in 2019 [19] and, the penetration rate of Twitter was 31% in 2020 for South Korean [21]. Thus, there should be at least 15.872 million Twitter users in South Korea. Previous studies on Twitter bots stated that up to 15% of Twitter accounts are bots, not people [22]. Thus, at least 18.2 million South Korean accounts (including the South Korean bot accounts) were expected to be scrapped. The figure in our results (18.3 million South Korean users by the twenty-sixth batch) was slightly larger than that we deducted from the statistics.

Then, to further validate our datasets, we devised a tweet matching validation approach to help validate the completeness of the tweet datasets. The basic idea of this approach is to compare the number of tweets that include a certain hashtag or keyword within our collected tweet dataset with the results that we could obtain from the Twitter firehose API by searching the same hashtag or keyword. For example, in our case, we aim to compare Korean language hashtag tweets in our collected timeline dataset with the Twitter firehose API results of the same hashtag. Twitter firehose API guarantees full access to the tweets that match the search criteria. Searching certain keywords on the Twitter firehose API and restricting the period to 2019 would provide 100% of the tweets containing the keywords in 2019. Meanwhile, our data collection strategy aims to reach the whole population of South Korean users who had tweeted in 2019. Thus, theoretically speaking, every tweet in the Korean language in 2019 should be found in our tweet results.

The specific validation procedure included three steps. In the first step, we extracted a list of popular Korean language hashtags from the tweet timeline dataset that we had scraped. In the second step, we collected all the tweets containing the popular Korean hashtags in 2019 from a commercial data analytics platform, Crimson Hexagon. The platform could get access to the Twitter firehose API and provide all the tweets by keyword searching. Then, in the third step, we examined the differences between the hashtag search results from Crimson Hexagon and the timeline tweet data in our network sample.

First, we identified the top 5,000 popular Korean language hashtags from our datasets. Surprisingly, we found that most of these hashtags were related to K-pop celebrities, K-pop events, and K-pop culture products. As K-pop culture is extremely popular and influential across the world [23], hashtags relating to K-pop culture could be heavily contributed by both South Korean fans and fans from other countries. Thus, we manually excluded K-pop related hashtags and identified 88 Korean language hashtags that were related to South Korean social events or everyday life, such as religion, food, domestic location, and domestic events. We expected that these hashtags were mainly contributed by South Korean users. The hashtags and English translations by Google Translation appear in Table 2.

**Table 2:** Comparison between hashtag searching results via Twitter firehose API and our datasets.

Hashtags	English translation of hashtag	Number of tweets downloaded via CH	Number of tweets included in our dataset	Number of overlapping tweets for two datasets	Coverage rate of our dataset on CH results
KBS뉴스	KBS News	46271	45565	40862	88.31%
사회뉴스	Social news	35809	34928	33096	92.42%
무료성경공부신청	Free Bible Study Application	35161	34339	29978	85.26%
안전공원	Safety Park	34343	19962	18480	53.81%
황교안	Hwanggyoan	34025	34209	32621	95.87%
한기총해체	Dismantling	32867	32945	30138	91.70%
정치검찰아웃	Political Prosecution Out	29538	28538	27485	93.05%
신흥무관학교	Sinheung Military School	29035	26512	22885	78.82%
비혼여성의_삶	Unmarried Women’s_Life	27633	25194	23527	85.14%
제주	Jeju	27492	27640	26540	96.54%
김재범	Jaebum Kim	25944	25182	23153	89.24%
세계평화선언문6주년	World Peace Declaration 6th Anniversary	25911	28679	25129	96.98%
박근혜	Park-Geun-Hye	24368	23938	23252	95.42%
한국	Korea	24064	22427	20817	86.51%
반종교한기총	Anti-religious	23205	22250	21240	91.53%
한기총해체촉구	Call for dismantlement	21873	21307	20142	92.09%
그래미	Grammy	21241	16223	14430	67.93%
북한	North Korea	20844	19689	18720	89.81%
삼일절100주년	100th anniversary	20841	20406	18313	87.87%
청와대	Blue house	19133	18890	18006	94.11%
한기총규탄대회	Korean Air Condemnation Contest	18421	18497	16251	88.22%
광복절	Liberation Day	18210	21867	15403	84.59%
세월호	Sewol issue	18179	18255	17136	94.26%
평화	peace	17988	19210	17257	95.94%
미세먼지	fine dust	17627	16466	15430	87.54%
강제개종피해인권연대	Forced Conversion Human Rights Alliance	17597	17345	15589	88.59%
여행	Travel	16390	13850	13017	79.42%
뉴스기사_남성성별_표기운동	News article_male gender_notation movement	16190	14817	13572	83.83%
거짓교리	False doctrine	15688	17363	12637	80.55%
검찰	Prosecution	15440	15135	14117	91.43%
성경인강	Bible Ingang	14946	14578	13426	89.83%
한글날	Hangul Day	14485	13835	10652	73.54%
광화문	Gwanghwamun	14108	14211	12655	89.70%
법무부	Ministry of Justice	14034	13374	12639	90.06%
법무부장관	Minister of Justice	13297	12725	12014	90.35%
버닝썬_미성년자_성매매_공론화	Burning Sun_Minor_Prostitution_Publicization	12000	12272	9791	81.59%
여권위조	Passport forgery	11852	11555	10310	86.99%
언론개혁	Press reform	11810	11480	10647	90.15%
속초산불	Sokcho Forest Fire	11171	11059	9102	81.48%
성신여대_미투	Sungshin Women’s University_Me2	11149	11040	8870	79.56%
광복74주년	74th anniversary of liberation	11128	10696	9061	81.43%
평창동계올림픽	Pyeongchang Winter Olympics	10966	9430	8421	76.79%
리얼돌수입허용판결규탄시위	Real Stone Import Permit Condemnation Protest	10943	19154	9062	82.81%
축구	Football	10928	10400	9471	86.67%
신천지경험	Sincheonji Experience	10683	10437	9364	87.65%
한반도평화통일	Peace on the Korean Peninsula	10561	11223	10224	96.81%
올림픽	Olympic	10290	8967	8128	78.99%
왜곡거짓보도	Distortion	10043	10739	9466	94.25%
촉법소년_미성년대상_교회성범죄_구속	Chokbeop Boy_Minority Award_Church Sex Crimes_Religion	10037	9557	7550	75.22%
속초_산불	Sokcho_ Forest Fire	9825	9043	7537	76.71%
신천지천안교회_피해성명발표	Sincheon Jicheon Church_Announcement of Damage	9721	10214	9163	94.26%
홍콩시민	Hong Kong citizens	9223	9642	7690	83.38%
서초동집회	Seocho-dong assembly	8921	8895	7890	88.44%
주민등록증	registration card	8768	10467	8734	99.61%
제주도	Jeju Island	8407	8527	7242	86.14%
부패한한기총	Corrupt Cold Gun	8379	9263	7218	86.14%
청계천로9월28일오후2시	Cheonggyecheon-ro September 28th 2pm	8119	15415	6833	84.16%
치킨	Chicken	7822	6661	5641	72.12%
세계인권선언	World Human Rights Declaration	7686	8224	6828	88.84%
무료성경	Free Bible	7672	7803	6733	87.76%
신천지교회	Sincheonji Church	7622	8264	7124	93.47%
울산	Ulsan	7365	6499	5485	74.47%
한국의신앙인들에게	For Koreans	7253	7569	6496	89.56%
장로교친일파	Presbyterian Pro-Japanese	7137	8017	5961	83.52%
진짜바로알자성경과신천지	Really, the Holy Scriptures and Shincheonji	7093	7817	6669	94.02%
천안기독교총연합회	Cheonan Christian Federation	6728	7332	6257	93.00%
강간카르텔유착수사규탄시위	Rape Cartel coalition investigation	6163	6166	4689	76.08%
신천지봉사	Sincheonji Service	5968	7060	5597	93.78%
종교연합사무실	Religious Union Office	5818	6886	5700	97.97%
피씨방	PC room	5766	4534	3608	62.57%
인권유린한기총	Human Rights Violations	5696	6360	4467	78.42%
미술관투어	Museum Tour	5549	4335	3446	62.10%
능욕	Rape	5548	5830	4368	78.73%
한국기독교부패알림	Korean Christian Corruption Notice	5405	6023	4795	88.71%
여성에게_정치권을	Political Rights to Women	4900	5479	3799	77.53%
신천지예수교	Sincheonji Jesus Bridge	4782	5894	4273	89.36%
온라인성경공부	Online Bible Study	3911	4502	3425	87.57%
대학교졸업증명서위조	Forgery of university graduation certificate	3798	3982	2696	70.98%
신천지수료식	Sincheonji completion ceremony	3602	3965	2674	74.24%
세계는신천지로	The world is Shincheonji	3491	4255	3059	87.63%
운전면허증위조	Driver’s license forgery	3427	3281	2044	59.64%
신천지_수료식	Sincheonji_Completion Ceremony	3160	3573	2410	76.27%
국가기술자격증위조	National technical qualification forgery	3073	3323	1975	64.27%
kbs검찰내통	kbs prosecutor’s office	3001	3754	2733	91.07%
와보라	Come on	2999	3870	2782	92.76%
통장위조	Forgery	2874	2740	1467	51.04%
병원진단서위조	Forgery of hospital diagnosis	2643	3073	1608	60.84%
황교안계엄령	Hwanggyo An martial law	2242	2920	2000	89.21%
Average		13492	13407	11627	84.40%

In the second step, we collected all the tweets covering the Korean hashtags via the Crimson Hexagon platform. As shown in Table 2, the column labeled “Number of tweets downloaded via CH” represents the hashtag searching results via the Twitter Firehose API with the language restriction as Korean, and the column labeled “Number of tweets included in our dataset” represents the results from our timeline dataset. Of note, the Twitter Firehose API could provide both a summary number of hashtags and access to download all the related tweets. The total summary number of hashtags covers not only existing tweets but also tweets that have been deleted by users. However, these deleted tweets could not be accessed and downloaded through the Twitter REST APIs that we used for the data collection. Thus, to compare the results efficiently, the number of tweets via Crimson Hexagon shown in Table 2 represents the number of existing tweets that could be downloaded at the end of 2019.

Finally, we compared the Twitter Firehose API results with our datasets. Importantly, when scraping users’ timeline data, Twitter REST APIs only allow developers to download the latest 3,200 tweets for one user. Thus, we could not guarantee that we had downloaded all the timelines of users in 2019 in our dataset. The solution to this problem was that we turned to examine whether the author of the tweets from the Twitter Firehose API results was included in our 2019 active South Korean user pool. If the author was covered by our data collection procedure via 26 batches of the snowball sampling approach, then the tweets were identified as included in our dataset. The results illustrated in Table 2 show that the coverage rates of our dataset range from 51.04% to 97.97%, with an average score of 84.4%.

**Fig 3:**
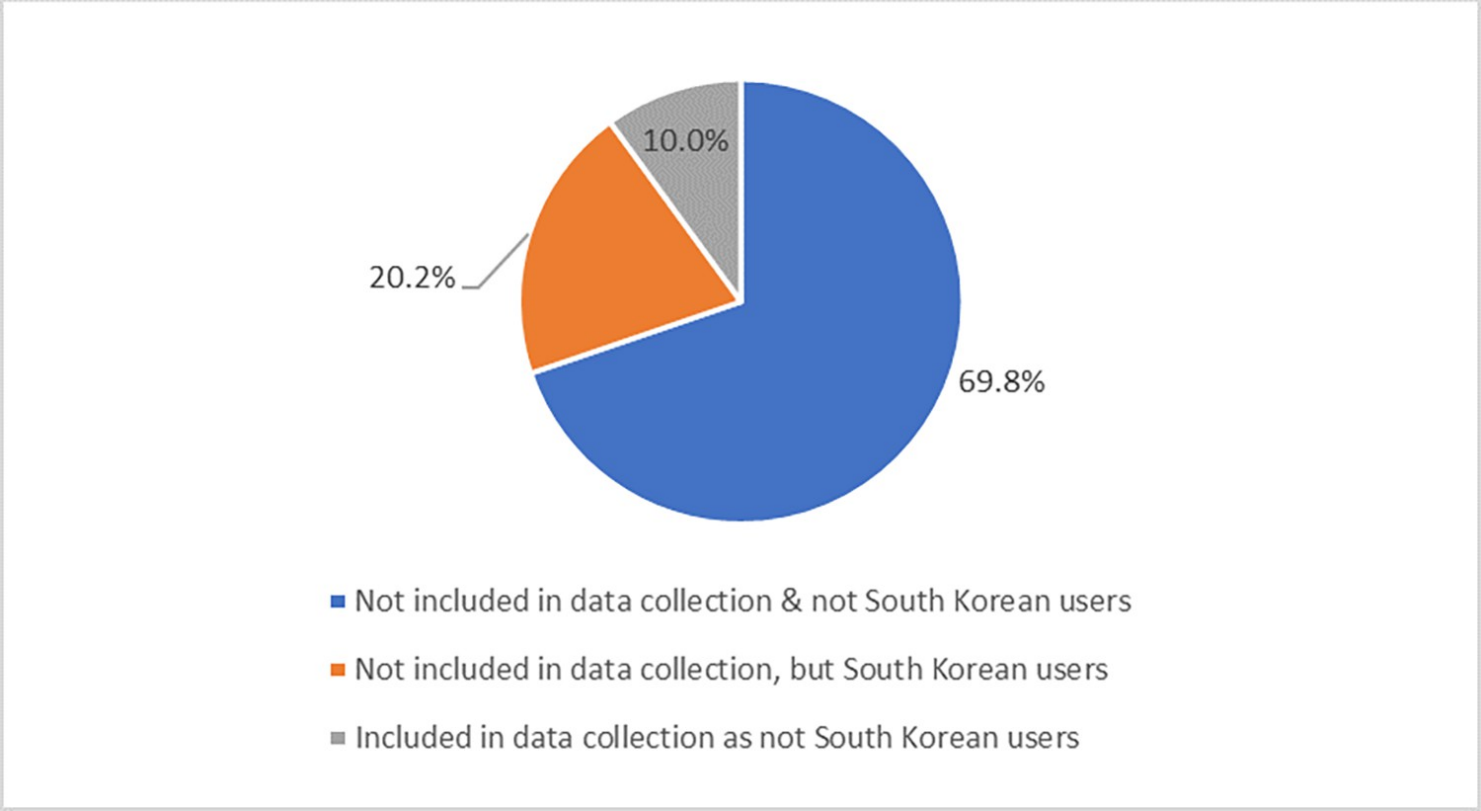
Reasons why tweets were not covered by our datasets.

We further looked into the tweets that were not covered by our datasets. Totally there are 165,989 tweets that were not covered by our data collection, and they are contributed by 20,608 Twitter accounts. Fig 3 shows the distribution of reasons why our datasets did not cover these accounts. We judged these accounts in two dimensions: whether these accounts had been covered in our snowball sampling procedure and whether these accounts showed any hints of nationality in their Twitter profiles. According to the results, 69.8% (14,380) of these accounts had neither been included in our sampling procedure nor showed any traces of South Korea in their profile information. Although these accounts had posted one or more tweets with Korean language hashtags, we could not guarantee the nationalities of these users. Since they neither wrote their Twitter names and descriptions in Korean characters nor set their locations within South Korea, they could possibly be foreigners tweeting on certain Korean issues. Furthermore, 10% (2,068) of the identified accounts were found to have been included in our snowball sampling procedure. However, during our data collection and nationality identification procedure, these accounts were labeled as not South Korean users due to a lack of South Korean traces in their profile information. Similar to the previous category of accounts, we could not guarantee their nationality since there was a chance that these accounts could be foreigners who were interested in Korean issues. Finally, there were 4,160 (20.2%) active South Korean users that should have been included in our datasets but were unfortunately not. Possible reasons could be that these accounts were relatively isolated in the South Korean user network. These accounts neither followed the most popular South Korean Twitter accounts from which we started our snowball sampling nor had any South Korean friends on Twitter (we would have covered them if they had any domestic friends). Given that we aimed to explore national patterns of the connected network structures, these isolated accounts should have minimally affected our results.

## Results

### Network structure

#### Power-law distribution in follower-followee network

**Fig 4:**
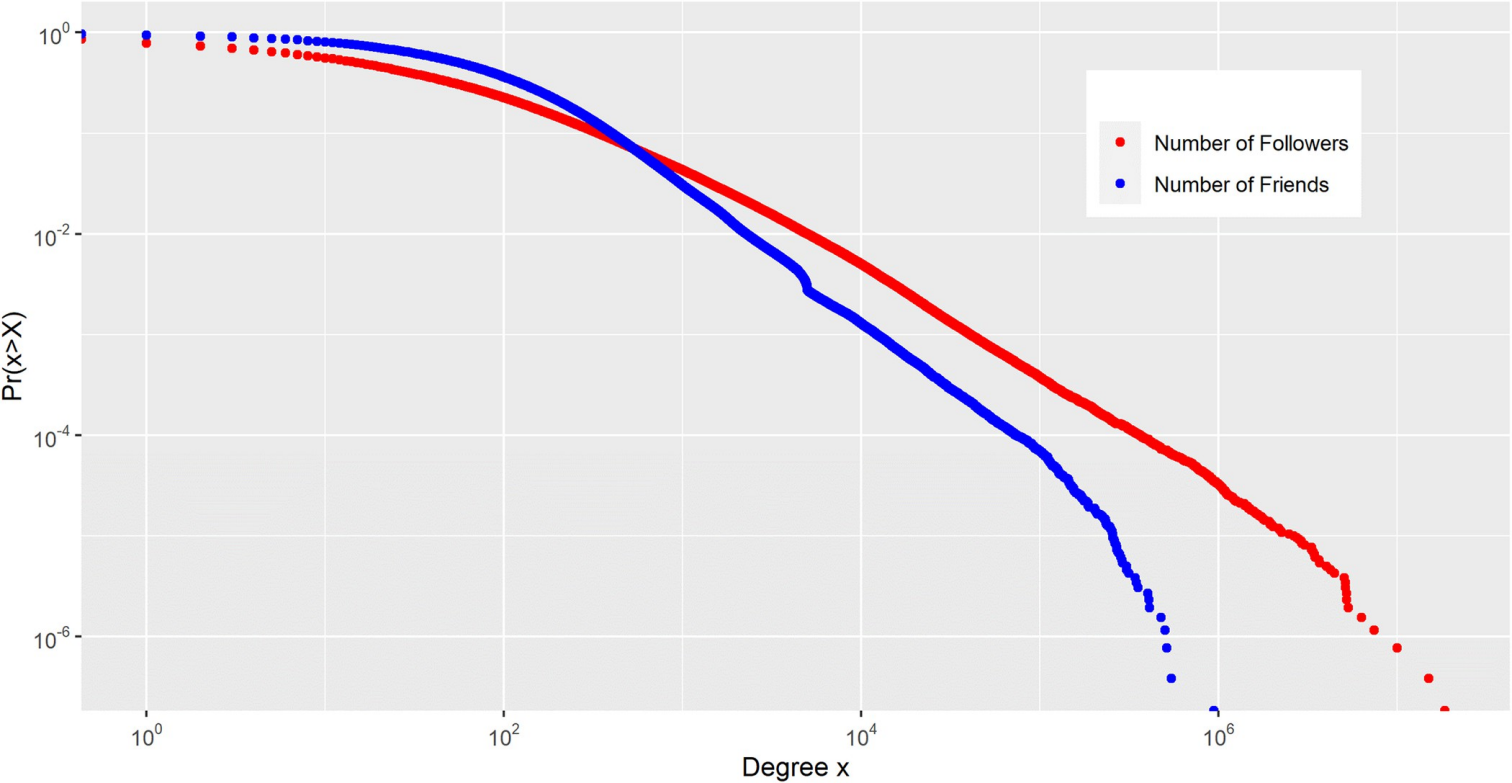
Log-log plot of the complementary cumulative distribution functions of the number of followers and number of friends.

We constructed the active South Korean Twitter user network in which nodes were active South Korean Twitter users, and edges were their following relationships. There were 2,309,959 nodes and 164,476,927 directed edges within the active South Korean Twitter network. Fig 4 displays the distribution of the number of followers and friends. The y-axis represents the complementary cumulative distribution function. Similar to previous literature [24, 25], our findings observe that both the number of followers (in-degree) and the number of friends (out-degree) on Twitter are unequally distributed with heavy tails, resembling power-law distributions (α_follower_ = 2.09; α_friend_ = 2.09).

#### Network attributes of the follower-followee network

In addition to the power-law distribution of follower-followee networks, some network attributes are frequently measured to depict the basic tie formation in the population-based following network in Twitter. Reciprocity, assortative mixing, and transitivity were calculated and compared with previous results of Twitter networks.

*Reciprocity* quantifies the likelihood of nodes to be mutually linked [26]. As reciprocity captures the symmetric form of interactions between two users, scholars believe the overall score of reciprocity could indicate how likely users take advantage of the platform’s social networking function and construct mutual interactions through the platform [27]. An early study in 2010 found that Twitter had a relatively low reciprocity of 22.1% [24], compared with other social media platforms with networking service (e.g., 68% on Flickr and 84% on Yahoo! 360) [28, 29]. The finding of low reciprocity for Twitter, as well as other findings on follower distribution and effective diameter, indicate that Twitter is more like news media rather than social media. Users on Twitter pay more attention to the news-related functions rather than constructing mutual friendships with others. However, a later study in 2014 overturned the previous conclusion that they found active user networks on Twitter actually had a much higher reciprocity level of 42% [30]. Our national network results find the reciprocity of the active South Korean network in our dataset was 37.86%. Compared to existing findings, the national Twitter network shows a moderate level of reciprocity, which indicates a moderate level of Twitter’s social function usage in the national network.

*Assortative mixing* indicates the preference for nodes in a network to link with other similar nodes [30]. Degree assortativity was regarded as a fundamental attribute to distinguish the social networks from other types of large-scale networks [31], thus it is one of the essential measures to help identify the social function of a network. In most undirected social networks, a typical degree assortativity ranges between 0.1 to 0.4. Previous literature found the directed networks (e.g., Twitter, Google+, etc.) have relatively low assortative scores. Existing reports of assortativity for Twitter range from neutral to quite disassortative (-0.053 to -0.88) [32]. The assortative measure of our national network is -0.0455, indicating a relatively moderate level of node homophily in the South Korean Twitter network.

*Transitivity* (also called clustering coefficient) measures the extent to which nodes in the network tend to cluster together and form closed triplet relationships. Research on social dynamics pays specific attention to the formation of friend-of-a-friend relationship [33], because the overall transitivity score reveals the existence of tightly connected communities (clusters, subgroups, cliques, etc.) and may influence the stability and development of the network in the long term [34]. We measure the transitive clustering coefficient for the directed network of Twitter. The score (tcc = 2.58%) is a bit higher than previous findings (1.9% in [35]). This indicates that the active Twitter user network of South Korea are more likely to cluster together and form connected triangle relationships.

#### Six degrees of separation

**Fig 5:**
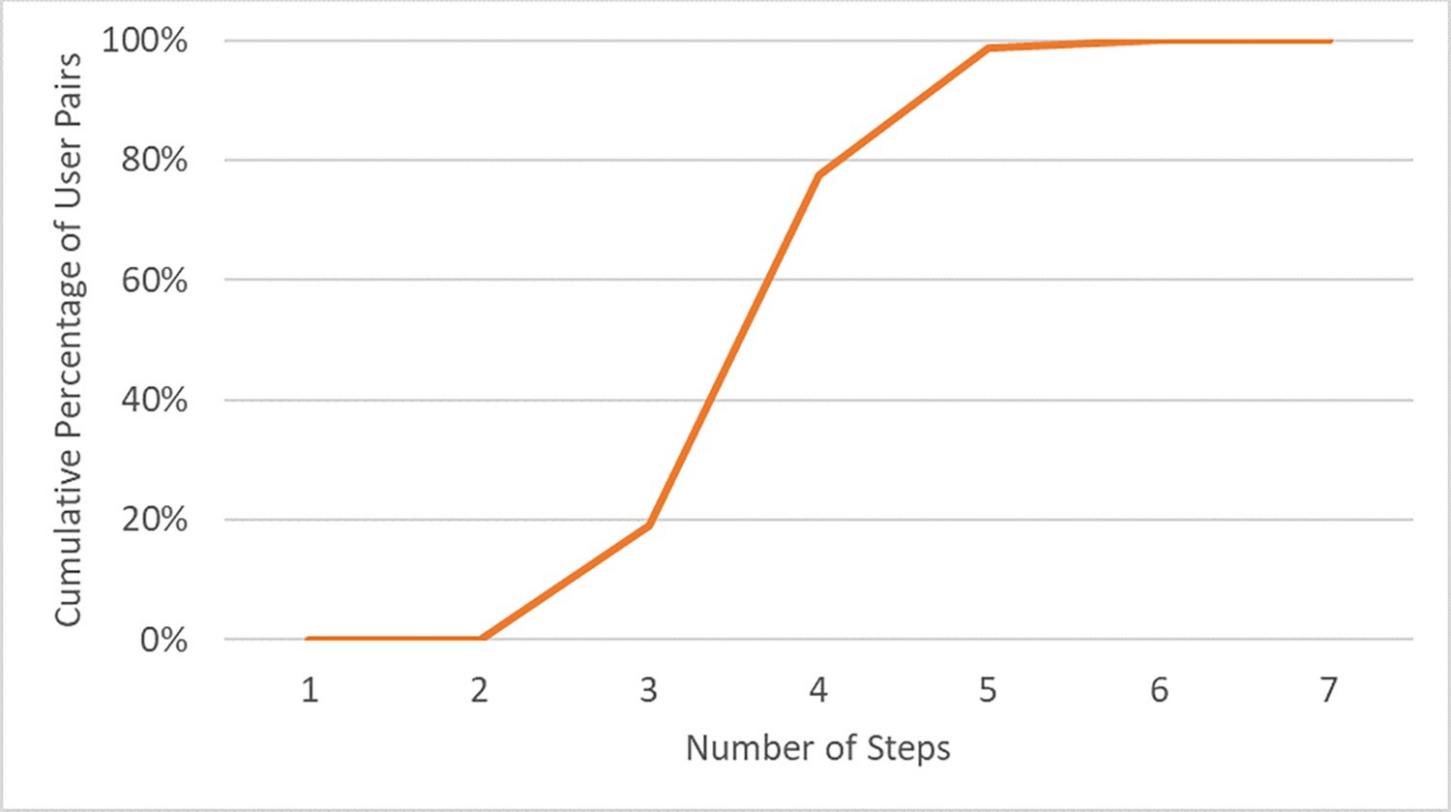
Cumulative percentage of user Pairs by number of steps.

The notion that people are connected through “six degrees of separation” is often used as a synonym for the idea of the “small world” phenomenon [36]. Degree of separation has been re-examined for many online networks, for example, e-mail networks [37], instant message networks [38], and webpage networks [39]. Previous literature suggests that the average path length of online social networks ranges from 3.43 to 4.47 [40–42]. In particular, users on Twitter are connected with an average degree of separation of 4.12 [24]. Our findings are similar to previous studies. Fig 5 shows that the average path length is 4.05, and 98.8% of user pair distances fall in five steps. The average path length is quite short considering the size of national Twitter network. That indicates the national network of Twitter users has a relatively close connectedness and exhibits some small-world properties.

### Usage

#### 80/20 rule of content generation

The 80/20 rule, also called the Pareto principle, originally refers to the phenomenon that approximately 80% of the land in Italy belonged to 20% of the population [43]. The rule was later generalized to both ownership, productivity, and other behaviour patterns in previous research on business, epidemic, and social media. For example, a small proportion of an organization’s marketing units often generates a large proportion of the profits [44]; 80% of epidemic transmission is accounted for by 20% of individuals [45]; more like a 76/20 proportion in UGC production on Internet [46], etc. Concerning the productivity on social media in particular, previous literature observed a power-law distribution with an exponent of about 1.92 on Twitter [47].

**Fig 6:**
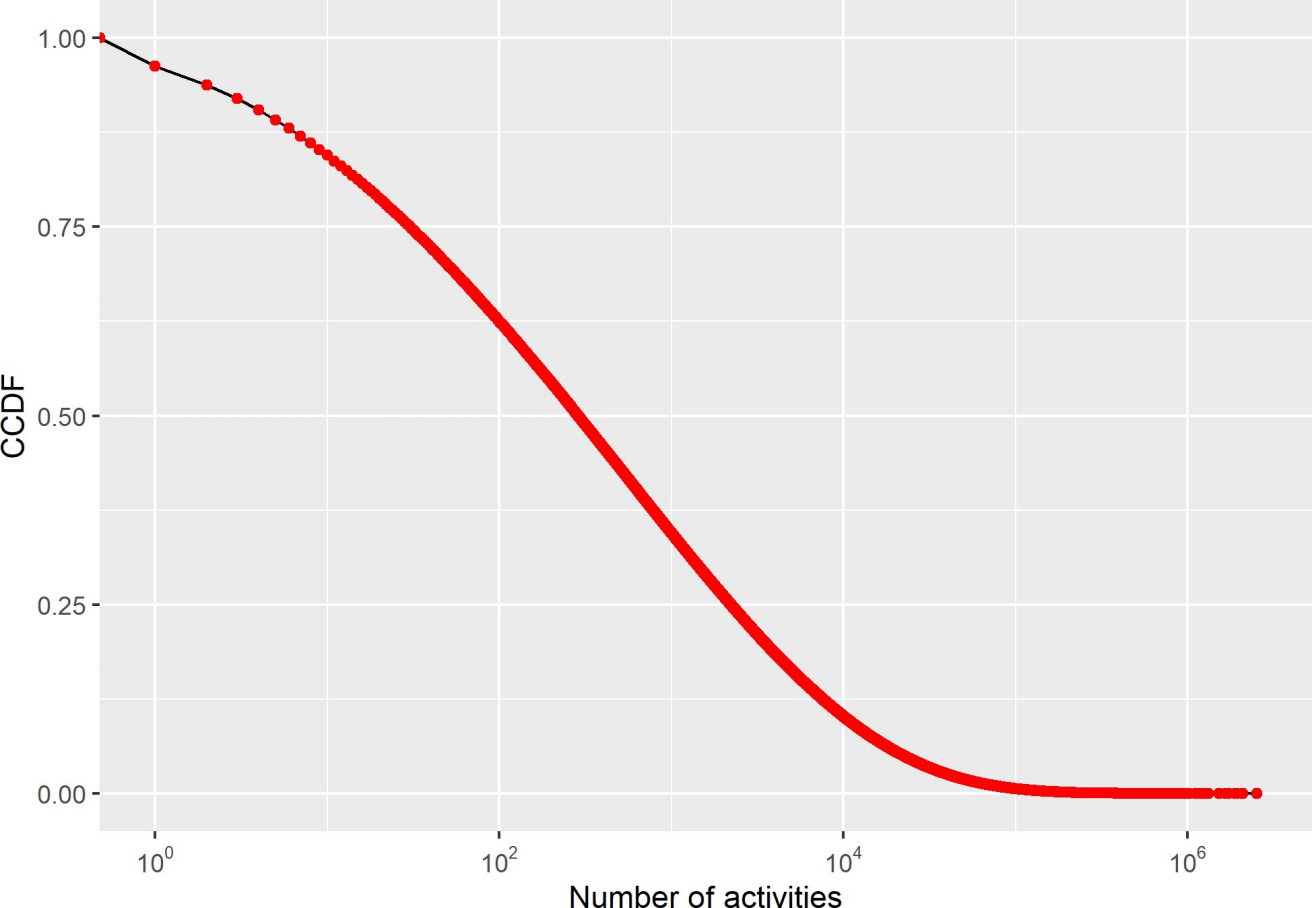
Semi-log plot of the complementary cumulative distribution functions of the number of tweets per user.

**Fig 7:**
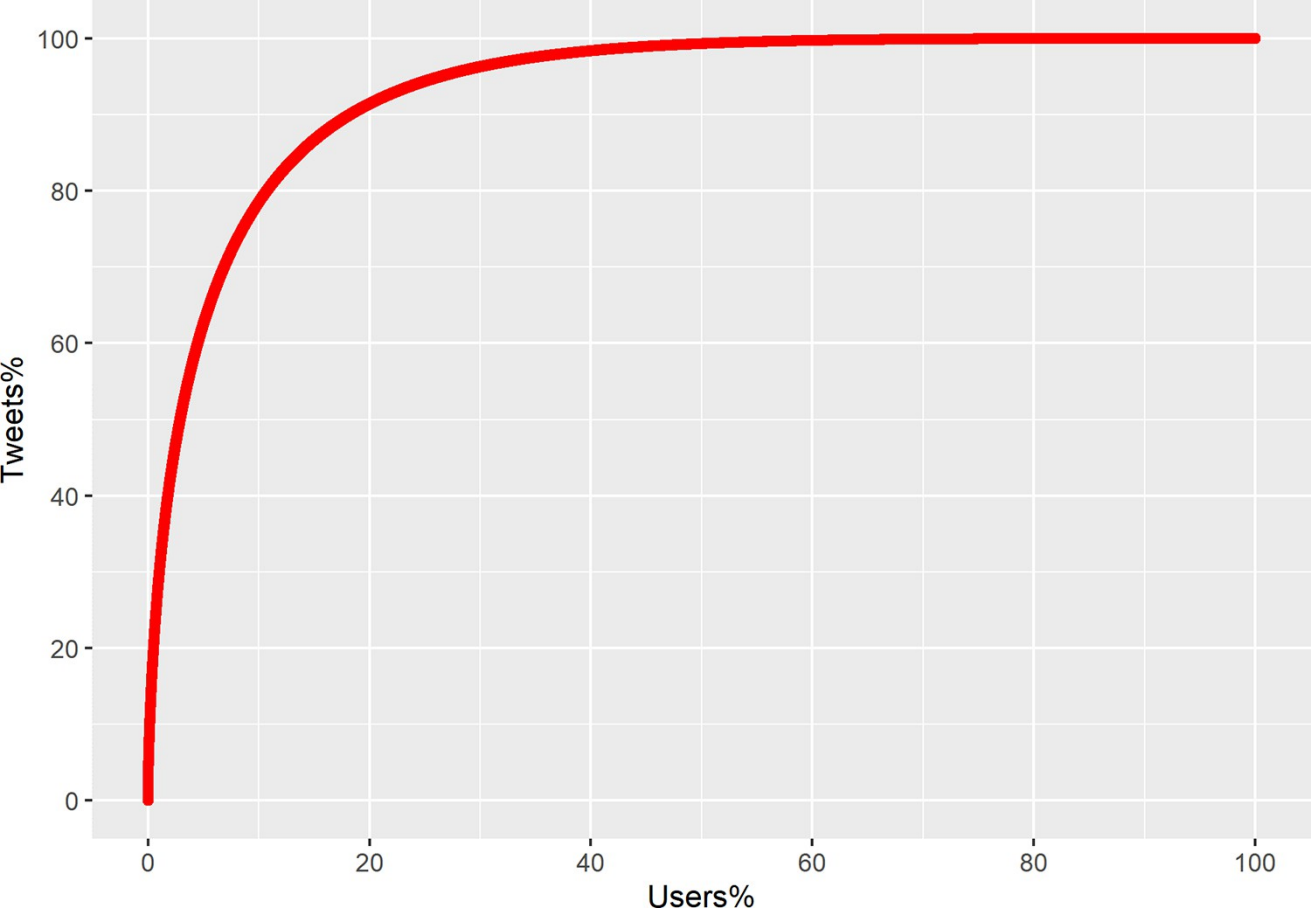
Cumulative percentage of tweets created by cumulative percentage of users.

Our results could not replicate the power-law findings on Twitter. Fig 6 illustrates that the distribution of the number of tweets per user follows a resemble exponential distribution than a standard power-law distribution. However, we did observe an approximate 80/20 rule based on Fig 7. About 20% of users created 92% of tweets, or 80% of contents are posted by 10% of users. More than half of the users do not make any obvious contributions to online content production.

#### Originality, sociability, and syntactic use

In previous literature, several syntax records and interaction features are frequently mentioned to describe how users post tweets: retweet, reply, hashtag, and URLs. They are commonly related to the research focus of information originality [48], sociability [49], and syntactic usage [50]. First, South Korean ranks in the forefront of retweet rate among multiple countries or cultural communities. The proportion of retweets in Korean language is 78% in 2018, lower than Thai (96%), but significantly higher than English (40%) and the representative random Twitter dataset (22.4%) [25, 51]. In our dataset, the percentage is 58%, which indicates a relatively low originality rate of South Korean Twittersphere compared to other cultures’.

Reply and @mention functions have been commonly regarded as indicators for conversations in Twitter [25]. The estimated proportion of replies and mentions in Korean-language tweets (ranging from 40% to 59% for replies and 73% for mentions) were relatively higher than other language, e.g., English (29% for replies and 47% for mentions) and all language (24% for replies and 49% for mentions) [25, 52]. In our datasets, 15.4% tweets are replies and 68.9% tweets contains @-mentions. As @-mentions could be caused by both retweet and reply-to. After excluding these, 2.1% tweets mentioned other users’ ID.

In terms of syntactic usage, previous research found there is a relatively moderate level of URLs (17%) and hashtag (11%) use in Korean language tweets, lesser than English language tweets (25% for URLs and 14% for hashtags), but similar to all language tweets (21% for URLs and 11% for hashtags) [52]. Our findings suggest that 14.7% Korean language tweets contain URLs and 23.8% tweets contain hashtags.

Overall, our findings indicate that there is a low-level originality (excluding retweets and replies: 26.3%) and a low-level sociability (reply+@mention: 17.5%) for South Korean users on Twitter. Twitter serves more like an information sharing site rather than social interactional platform for South Korean community.

#### Circadian rhythms

**Fig 8:**
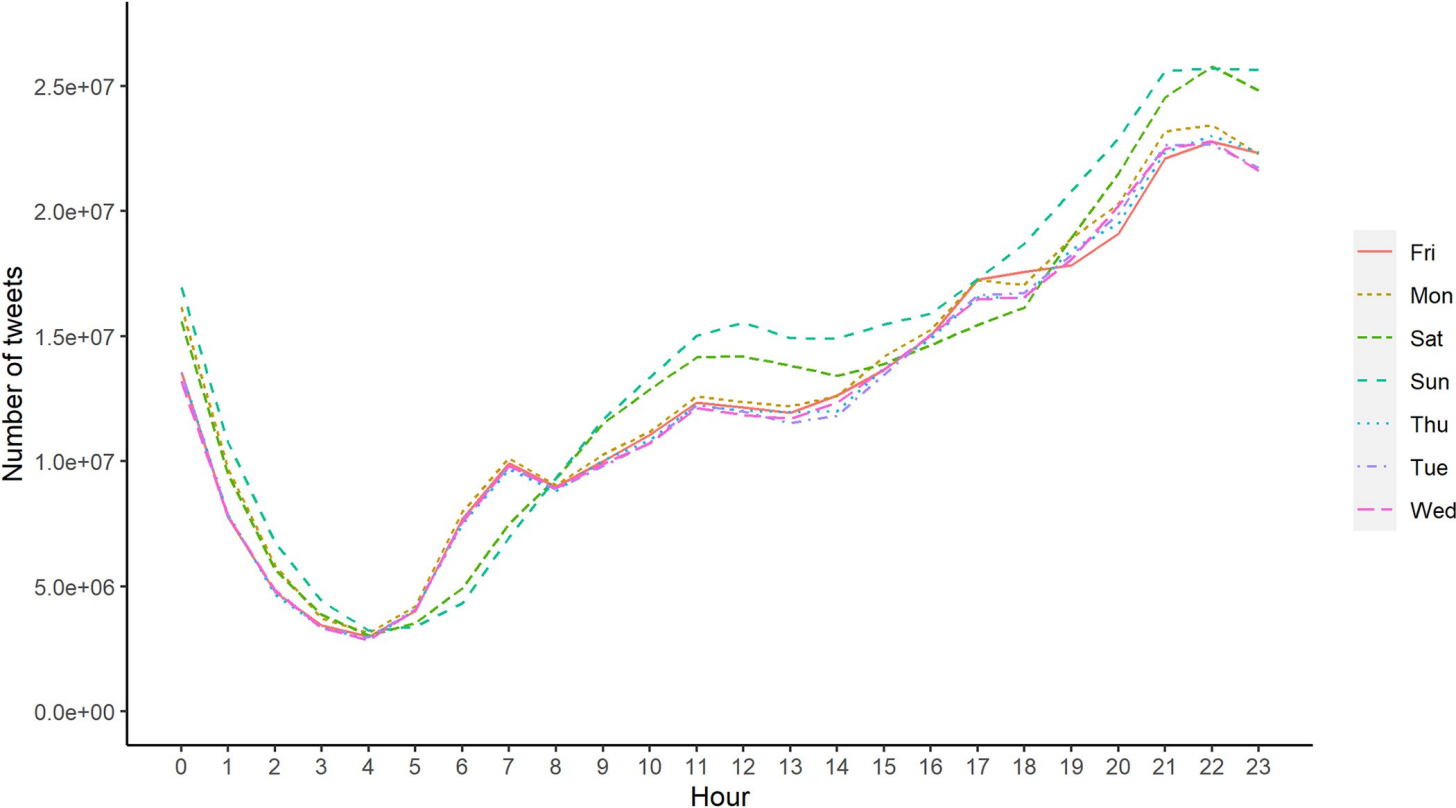
Number of tweets posted by hour of the day and day of the week.

Consistent with previous findings on digital device and social media usage, Twitter message activity was found to follow the daily and weekly circadian rhythm [53]. Twitter usage starts to rise in the morning, continue to increase during the daytime, and peak in the evening [25]. Besides, previous research observed different usage patterns between weekdays and weekends for individuals with different demographic characteristics [54]. Our results are consistent with previous studies on daily circadian rhythm. Fig 8 illustrates that Twitter messaging activity in South Korean network reaches the peak around 9:00 pm for both weekdays and weekends. Besides, we also observe that Twitter usage at weekends were lower in the morning, but higher at noon and night, compared to weekday activities.

#### Topics & social bots

To better understand the users’ profile and their topic attention, we further looked into their timelines. We conducted the unsupervised topic modelling approach on a random sample of 10,000 users’ timeline (more than 340k tweets). To better interpret the results, we firstly translated the tweets into English and applied the state-of-art topic modelling technique, BERTopic [55], to classify the topic. By manually interpreting the meaning of each topic, we classified all the tweets into 6 categories. Among these tweets, 30.1% were recognized as related to K-POP and celebrity activities, followed by fashion and art (23.3%), technology (16.5%), daily life (13.9%), game & sport (8.4%), and other uncategorized personal statements (7.8%).

Besides, we detected the average Twitter bot rate in active 2019 South Korean datasets using a 30,000 random user sample. Using a widely-used library BotometerLite [56], we found that 51.5% of accounts were identified as bots with a botlikelihood score of .5 or higher. The corresponding rate changes to 42.1% for a threshold of .6, 33.0% for a threshold of .7, and 21.7% for a threshold of .8. The bot rate is much higher than previous results for English-language tweets (13.2% [57] and 15% [22]). However, considering the culture difference of South Korean [58–60], our results are quite similar to previous results for Asian-language tweets (52% for Indonesia, 52% for Philipplines, 71% for Singapore, and 63% for Taiwan, with a BotHunter threshold of .5) [61].

## Conclusions and discussions

The current study evidenced a data collection strategy to scrap and validate a national-level Twitter network. To collect the population of active South Korean Twitter users, we conducted twenty-six batches of data collection by utilizing the snowball sampling method. Then, we proposed two approaches to help evaluate the validity of our dataset. The first one is to roughly compare with public statistics of Twitter usage in South Korean. Then, to further validate our datasets, we devised a tweet matching validation approach to help validate the completeness of the tweet datasets. After comparing the number of tweets that include a certain hashtag or keyword within our collected tweet dataset with the results that we could obtain from the Twitter firehose API by searching the same hashtag or keyword, we found a relatively good coverage rates of our dataset, with an average score of 84.4%. Finally, we re-examined a series of network patterns and social theories in the full social network of South Korean, including network attributes (network distribution, reciprocity, assortative mixing, transitivity, degree of separation, etc.) and usage patterns (originality, sociability, syntactic use, circadian rhythms, topics distribution, social bots, etc).

The study could bring practical contributions to network analysis research in two ways. First, this study evidenced a feasible and flexible strategy to scrap the national Twitter networks and devises two validation approaches to verify the completeness of the collected network. Although we only demonstrate one case, it could be flexibly applied to many other nations or ethnic groups. For example, the proposed approach could be directly utilized to other nations that use their own unique language as the mainstream communication language, e.g., Japan, Bulgaria, etc. The strategy would largely flourish the data collection pool of population-level social networks and further develop the research of network analysis in digital media environment.

Second, national online social networks could serve as a reference to help validate existing network sampling methods. Probability sampling in network science are difficult due to the lack of a sampling frame for the population structure of social media users [7]. Many prevailing sampling methods (e.g., random walks) were proposed to conduct ego selection, where the data collection starts with some randomly selected seed nodes and continues with the random selection of links from the seeds [62]. However, some of these methods (e.g., the initial version of the random walk) have been questioned to be systematically biased toward the hub nodes, as it cannot solve the self-selection bias inherent in the link structures of a network [63, 64]. In this case, large-scale population-based networks are in crucial needs to help validate the network sampling biases. The national social network is one of the optimal labelled reference for network sampling validation, as it is the largest community-level networks with the basis of a common territory, language, and culture.

Finally, current study could also provide theoretical implications to existing social science literature. We re-examined classical network patterns and theories on the national social network of South Korea and found new patterns on online full population network. For example, our works show that the national network of South Korea has a relatively low degree of separations, which indicates a close connectedness and “small-world” properties for the South Korean society. Future studies could further examine the connectedness of other population-level social networks and estimate how the extents of “small-world” effects vary among different societies.

## Limitations and future studies

Our study is subject to some limitations. First, we note that our dataset could still contain false positives and omit false negative: it would also match false-positive accounts such as foreigners who lived in South Korea. In addition, there may be South Korean users never using Korean language on Twitter. We admit this existing situation and this is the common problem for all so-called national-level Twitter networks in previous literature. This study have utilized two validation methods to help minimize it.

Another obvious limitation is about the language challenge. As non-Korean researchers, we employed English translations of sampled tweets in Korean to conduct the topic modeling. The results might be more correct if applying BERT to tweets in Korean without first translation. Future studies could apply native language packages to help validate our results.

Besides, in this study, we only considered a single social platform for collecting the population-level networks. Future directions could replicate or further develop the approach to other social networking sites. Finally, current study only examines some descriptive patterns and findings on the population-level social network. Future studies would continue to investigate on other correlational or causal patterns of the population network, e.g., the causes of population networks formation, the relationship between network structures and functionalities, etc.

## Supporting information

S1 Appendix (DOCX)
