# Supplementary material for: Census of Twitter users: Scraping and describing the national network of South Korea
Source: PLoS One. 2022 Nov 17;17(11):e0277549. doi: 10.1371/journal.pone.0277549 (PMC9671467; doi:10.1371/journal.pone.0277549)
Supplement: S1 Appendix — (DOCX) [file pone.0277549.s001.docx]

Table 1. Datasets used for testing propositions and measurements

| Level | Proposition | User profile | Following relationship | User timeline | Measurements |
| --- | --- | --- | --- | --- | --- |
| Network Structure | 1. Power-law distribution in follower-followee network | √ | √ |  | Distribution of user’s number of followers and followees |
|  | 1. Reciprocity | √ | √ |  | Likelihood of nodes in the network to be mutually linked |
|  | 1. Assortative mixing |  | √ |  | Degree Assortativity |
|  | 1. Transitivity |  | √ |  | Transitive clustering coefficient |
|  | 1. Six Degrees of Separation |  | √ |  | Average path length of the network |
| Usage | 1. 80/20 Rule of content generation | √ |  |  | Distribution of user’s post production |
|  | 1. Originality |  |  | √ | Rate of retweets in total posts |
|  | 1. Sociability |  |  | √ | Rate of replies and mentions in total posts |
|  | 1. Syntactic use |  |  | √ | Rate of URLs and hashtag in total posts |
|  | 1. Circadian rhythm |  |  | √ | Users’ daily circadian rhythm for each day of the week |
|  | 1. User category | √ |  | √ | Classify users into eight categories according to their timeline texts. |
|  | 1. Twitter bots | √ | √ | √ | Identify users’ likelihood to be twitter bots using Botornot. |
